# A Patient with Clavicle Fracture and Recurrent Scapular Winging with Spontaneous Resolutions

**DOI:** 10.1155/2012/603726

**Published:** 2012-11-01

**Authors:** Kendra E. Keenan, John G. Skedros

**Affiliations:** ^1^Utah Orthopaedic Specialists, 5323 South Woodrow Street, Suite 200, Salt Lake City, Utah 84107, USA; ^2^The University of Utah Department of Orthopaedics, Salt Lake City, Utah 84108, USA; ^3^Intermountain Medical Center, Salt Lake City, Utah 84107, USA

## Abstract

Injury to the long thoracic nerve with resulting serratus anterior palsy is a typical cause of medial scapular winging. We report a case of a 70-year-old female with scapular winging in the setting of a mildly comminuted midshaft clavicle fracture. The winging persisted for three months after the fracture, which became a nonunion. The winging spontaneously resolved prior to open reduction and internal fixation of the nonunion. The winging recurred after this surgery. The recurrence was attributed to transient irritation and/or inflammatory neuropathy of the brachial plexus caused by the surgical manipulation. This second episode of winging again spontaneously resolved. There are few reported cases of scapular winging in the setting of a clavicle fracture and only one case of recurrent scapular winging. In that case, which was in the setting of an acromioclavicular joint separation, the second episode of winging required long-term use of a brace. By contrast, our patient did not require bracing because the recurrent winging spontaneously resolved, making this a novel case. This case is important because it illustrates that recurrent scapular winging can occur, and spontaneously resolve, in the setting of a mid-shaft clavicle fracture after subsequent reconstruction of a fracture nonunion.

## 1. Introduction 

Damage to the long thoracic nerve is a classic cause of medial scapular winging [1, 2]. This injury, which results in palsy of the serratus anterior muscle, can be caused by a variety of acute trauma events and chronic repetitive activities. In some cases, the winging is not associated with electrodiagnostic evidence of injury to the long thoracic nerve or cervical nerve roots that contribute to it (C-5, C-6, and C-7) [1–3]. With respect to fractures of the shoulder girdle, there are reports of medial scapular winging in association with (1) malunions and nonunions of acromion fractures [2, 4], (2) malunions and nonunions of clavicle fractures [1–3], (3) clavicle fractures with other associated injuries [3, 5], (4) glenoid fractures [1], and (5) malunions of scapular body fractures [2]. Although our review of the English literature revealed a few cases of ipsilateral medial scapular winging in the setting of clavicle fractures prior to being considered nonunions [3, 5], we could only locate one case describing recurrent winging. In that case the patient did not sustain a clavicle fracture, but had an acromioclavicular separation and required a brace to keep working because the winging did not resolve after the recurrence [4]. 

We report the case of a patient who sustained a closed and minimally comminuted mid-shaft clavicle fracture after a ground-level fall which developed ipsilateral scapular winging that became obvious and clinically significant when she started physical therapy prior to sufficient healing. The winging resolved completely in three months, only to recur after the surgical reconstruction of the nonunion that developed. This second episode of winging also completely resolved three months later. Additional unusual aspects of this case include (1) electrodiagnostic studies revealing evidence of mild injuries to the brachial plexus in addition to the long thoracic nerve and (2) there were no fractures or significant injuries to the ribs or scapular body or to the acromioclavicular, sternoclavicular, and glenohumeral joints. 

## 2. Case Report

**Figure 1:**
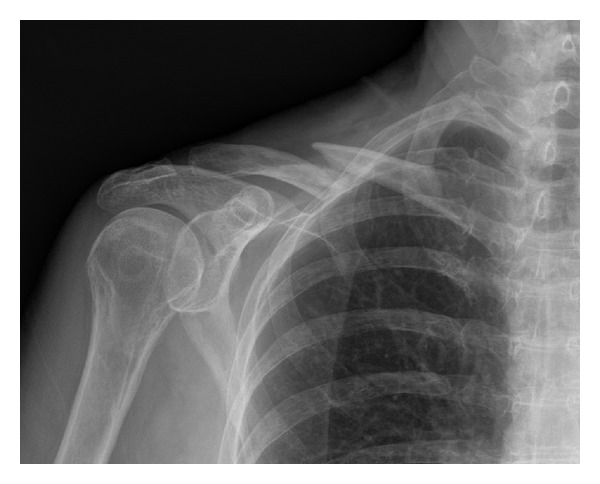
Radiograph from date of injury showing the right mid-shaft clavicle fracture. A 2.5 cm fragment is displaced posteriorly, but is not seen well in this radiograph.

A previously healthy 70-year-old right-hand-dominant female was seen in our clinic three months after sustaining a closed and minimally comminuted mid-shaft fracture of the right clavicle, which resulted from a ground-level fall (Figure 1). She tripped on a raised edge of a concrete driveway while walking and fell directly onto the lateral aspect of her shoulder without extending out her hand. There was no head or neck trauma, no rib fractures, and no significant trauma to the other extremities. She was initially under the care of another physician who had prematurely started her on a physical therapy program (including active overhead motion) one month after injury even though there was no radiographic evidence of callus formation. This premature initiation of physical therapy contributed to her report of severe pain and clicking/catching sensations at the fracture site. She also complained of numbness and tingling radiating along the ulnar forearm and burning sensations at the inferior aspect of the scapula as well as radiating distally to the upper anterior forearm. 

Physical examination showed swelling in the mid-clavicular area and crepitance with attempted shoulder motion. Medial winging of her right scapula was obvious and was viewed from behind while the patient was pushing against a wall. Strength with grip, finger abduction, and thumb extension was decreased (4/5) on manual testing. A nerve conduction study and electromyographic evaluation (NCS/EMG) showed evidence of (1) mild brachial plexopathy with involvement of the upper trunk and axillary nerve, in addition to a slightly decreased ulnar motor response and (2) long thoracic nerve injury. The NCS/EMG showed reduced axillary nerve amplitude. There were also motor unit changes in the muscles innervated mainly by the long thoracic and also by the axillary, radial, and ulnar nerves. Based on NCS/EMG criteria, the overall prognosis for recovery was considered good. Recommendations included stopping physical therapy and starting daily treatments with an ultrasound-based bone growth stimulator (OrthoLogic, dj Orthopedics, LLC, Vista, CA, USA). 

Several outcome surveys were completed at each visit (Table 1). Four months after the fracture occurred, pain had increased at the fracture site, but the radiating burning sensations had abated. She also continued to complain of clicking sensations at the fracture site with attempts at grooming and changing clothing. Strength with grip and finger abduction showed improvement. Scapular winging persisted. Radiographs showed early callus formation at the medial aspect of the fracture. The bone growth stimulator was continued. One month later, the winging was no longer present. 

**Figure 2:**
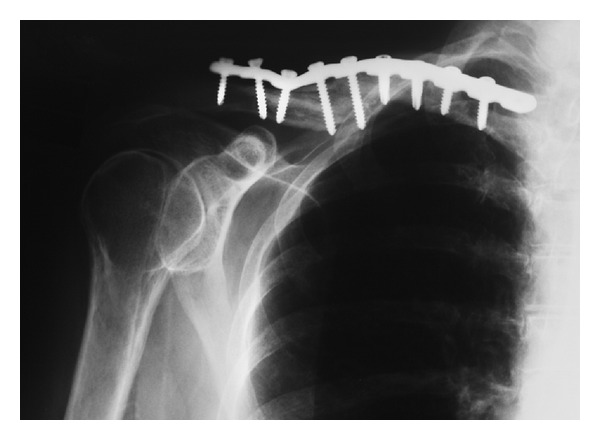
Radiograph of the right clavicle with fracture fixation plate and screws. The inferior prominence of the bone may have reaggravated the brachial plexus injury. Revision surgery was not required.

Radiographs six months after the fracture showed a well-established nonunion. Treatment was open reduction and internal fixation with bone grafting (Figure 2). Although the surgery was otherwise uncomplicated, the scapular winging recurred. The winging spontaneously resolved within three months after surgery and did not recur. 

**Figure 3:**
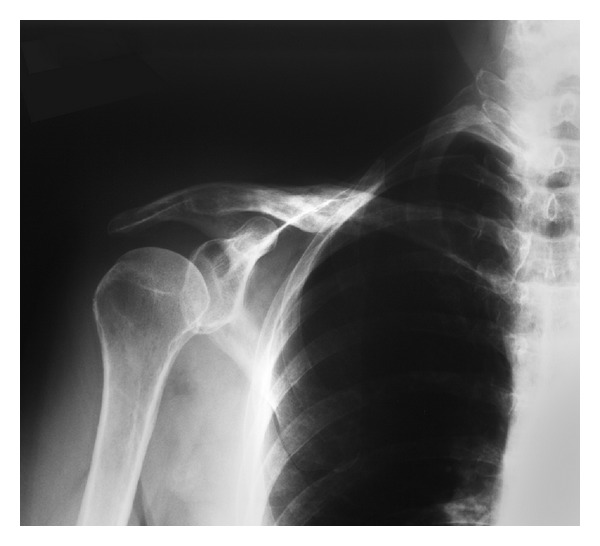
Radiograph of the healed right clavicle fracture after hardware removal.

Nine months after surgery, the patient underwent hardware removal because of local discomfort from clothing. Radiographs taken fourteen months after fracture fixation surgery and six months after hardware removal (20 months after injury) show complete healing of the clavicle fracture (Figure 3). She reported no pain and the shoulder examination was normal. Strength in grip, finger abduction, and thumb extension was normal. All outcome measures showed improvement. Additional improvement was also reported by surveys that were mailed at 20 months after fracture fixation surgery (26 months after the fracture originally occurred) (Table 1). She reported complete satisfaction in a telephone conversation at 4.5 years after fracture fixation surgery (five years after the fracture occurred). 

## 3. Discussion

Although medial scapular winging has been reported to occur in association with nonunion and malunions of shoulder girdle fractures [1, 3, 5, 6], our review of the English literature only revealed one case of recurrent scapular winging [4]. In this case, the patient had an acromioclavicular separation without fracture. Because the second occurrence of winging did not resolve, that patient required a brace for his employment as a manual laborer. This contrasts with our patient who had two episodes of scapular winging, both with spontaneous resolution during the period of fracture treatment. 

Clavicle fractures account for 2.6 to 4% of all fractures, and of those approximately 80% are mid-shaft clavicle fractures [7, 8]. The association of scapular winging with a clavicle fracture is uncommon. For example, in 1,288 reported cases of clavicle fracture (excluding nonunions), only four cases of scapular winging were reported [1, 3, 5, 6, 9–13]. This suggests that the prevalence of scapular winging with mid-shaft clavicle fractures is less than 0.05%. 

By contrast, brachial plexus injury (without scapular winging) is more commonly associated with clavicle fractures. For example, of 111 cases of mid-shaft clavicle fracture reported by the Canadian Orthopaedic Trauma Society [7], eight of their operative patients and seven of their non-operative patients had transient brachial plexus symptoms. Brachial plexus injury has also been reported in association with clavicular malunions and nonunions [14–18]; however, scapular winging has only been reported in a few cases [1, 3, 5, 6]. 

Barbier et al. [16] report a case of a 32-year-old male who sustained a comminuted mid-shaft clavicle fracture from a fall and suffered from continuous pain in the arm and shoulder from a 2.5 cm bone fragment that was pressing directly on the proximal part of the posterior cord. The symptoms resolved after removal of the fragment. Matz et al. [19] report a case of a 21-year-old male football player who sustained a comminuted mid-shaft clavicle fracture. After the fracture healed with nonoperative treatment, their patient complained of weakness and dysesthesia. Electrodiagnostic studies showed compression of the lateral cord of the brachial plexus. Abundant callus was surgically removed, which alleviated all symptoms. Rüst et al. [20] describe a young adult male who sustained a comminuted mid-shaft clavicle fracture after crashing on a bicycle during a triathlon. The musculocutaneous nerve was impaired and there was decreased sensation across the region of the anterior deltoid muscle. The fracture was treated acutely with an intramedullary nail. Although normal sensation eventually returned, the muscle atrophy did not improve. These observations, in addition to other similar reports [17], are important in the perspective of our case because they show that fragments of mid-shaft clavicle fractures can cause injury/irritation to deeper portions of the brachial plexus. 

Rasyid et al. [3] report a case of scapular winging in a 52-year-old female who sustained a comminuted mid-shaft clavicle fracture and ipsilateral second, third, and fourth rib fractures. She did not notice winging until two months after injury. The fracture was then treated with open reduction and internal fixation. There was no electrodiagnostic evidence of long thoracic or axillary nerve palsy and the winging resolved soon after surgery. Pearsall and Russell [5] describe a 16-year-old male wrestler that sustained a comminuted mid-shaft clavicle fracture and ipsilateral sternoclavicular joint subluxation when his opponent fell onto his shoulder. Three weeks later, scapular winging appeared and was associated with long thoracic nerve injury. The scapular winging had completely resolved nine weeks after injury. These cases contrast with our report because they were associated with rib fractures and/or additional injuries to the shoulder girdle.

Gonza and Harris [6] reported one of 14 cases of scapular winging in association with a clavicle fracture. Similar to our patient, all of these 14 patients were noted to have a burning sensation at the lower aspect of their scapula. Gonza and Harris [6] suggested that the probable cause of winging in the majority of their nonfracture cases was from compression of the long thoracic nerve against the second rib. Gregg et al. [4] offered an explanation for the mechanism of long thoracic nerve injury based on their 10 patients who developed scapular winging from non-fracture trauma to the shoulder girdle: (1) contralateral head tilt, flexion, and rotation and (2) elevation of the ipsilateral arm overhead. These combined events cause the long thoracic nerve to be significantly stretched (i.e., possibly >10%) because it is pulled anteriorly and medially while the serratus anterior moves posteriorly, laterally, and inferiorly. Our patient appears to have a different set of circumstances that contrasts with this mechanism of injury. 

Our interpretation is that our patient sustained a partial neuropraxia of the brachial plexus, which manifested distally as involvement mainly of the long thoracic nerve, and to a lesser extent, the axillary, ulnar, and radial nerves. This interpretation is consistent with her NCS/EMG findings and is more likely than an isolated injury to the long thoracic nerve [21]. The recurrence of the scapular winging supports the interpretation that the brachial plexus was primarily involved because of its proximity to the surgical manipulations required during fracture reconstruction. In this context, the mild shortening of the clavicle after reconstruction may have contributed to the recurrent neuropathy and scapular winging (Figure 2). Another possibility is that the patient developed an inflammatory neuropathy after surgery leading to the recurrent scapular winging, which resolved when the inflammation subsided. 

## 4. Conclusion

 Our patient presented with a unique case of medial scapular winging and spontaneous resolution prior to nonunion of her clavicle fracture and with spontaneous and permanent resolution of the recurrent winging after fracture reconstruction. We suspect that the winging was caused by injury the brachial plexus, which indirectly affected the long thoracic nerve. Our case is also unusual because there were no fractures or apparent significant injury to the ribs and scapular body, or to the acromioclavicular, sternoclavicular, and glenohumeral joints.

## Figures and Tables

**Table 1:** Outcome scores and physical examination data: visual analog scale (VAS) for pain, Western Ontario rotator cuff index (WORC), the American shoulder and elbow surgeons standardized shoulder assessment form (ASES), simple shoulder test (SST), disabilities of the arm, shoulder and hand (DASH) score, short form 36 (SF-36), and shoulder range of motion (ROM) measurements.

	Postinjury months (fracture repaired at 6 months after injury)						
	3 months after Injury	4 months after Injury	Surgery 6 months after injury	12 months after injury	Hardware removal 15 months after injury	20 months after injury	26 months after injury
Active forward flexion	70°	—		170°		170°	—
Winging	Yes	Yes		No		No	No
10 cm VAS pain on typical day (best = 0)	3.9	6.1		0.3		0	0
ASES score (best = 100)	47.2	—		90.2		—	—
WORC score (best = 0 (100%))	—	1619 (23%)		570 (73%)		192 (91%)	—
Simple shoulder test^†^ (best = 12 yes responses)	—	3		8		11	12
DASH score (best = 0)	—	77		17		2	4

Short form-36*							

Physical functioning	—	40.0		75.0		70.0	88.8
Role limitations due to physical health	—	0.0		50.0		75.0	100.0
Role limitations due to emotional problems	—	100.0		100.0		100.0	100.0
Energy/fatigue	—	75.0		70.0		80.0	90.0
Emotional wellbeing	—	80.0		88.0		88.0	76.0
Social functioning	—	50.0		100.0		100.0	100.0
Pain	—	22.5		67.5		67.5	90.0
General health	—	70.0		80.0		85.0	85.0

^†^Number of yes responses/number of questions (“yes” responses correlate with better shoulder function than “no” responses). Twelve “yes” responses are possible.

*All questions are scored from 0 to 100, with 100 representing the highest level of functioning possible. Aggregate scores are compiled as a percentage of the total points possible, using the RAND scoring table.
